# Association between Functional Capacity Decline and Nutritional Status Based on the Nutrition Screening Initiative Checklist: A 2-Year Cohort Study of Japanese Community-Dwelling Elderly

**DOI:** 10.1371/journal.pone.0166037

**Published:** 2016-11-08

**Authors:** Yumiko Sugiura, Yoshimi Tanimoto, Ayumi Imbe, Yuiko Inaba, Satoshi Sakai, Kanako Shishikura, Keiji Tanimoto, Toshiaki Hanafusa

**Affiliations:** 1 Medical Corporation Hatsunekai Sugiura Clinic, Kariya City, Aichi, Japan; 2 Departments of Internal Medicine (I), Osaka Medical College, Takatsuki City, Osaka, Japan; West Virginia University School of Medicine, UNITED STATES

## Abstract

**Aim:**

To assess whether nutritional status based on the Nutrition Screening Initiative Checklist is useful for predicting functional capacity decline in community-dwelling Japanese elderly.

**Methods:**

This two-year observational cohort study included 536 community-dwelling Japanese (65 years and older at baseline) who were independent in both activities and instrumental activities of daily living. Demographic attributes, chronic illness, lifestyle-related habits, nutritional status, functional capacity, and anthropometric measurements were assessed, with decline in functional capacity used as the outcome measure.

**Results:**

Subjects were classified into three groups as follows based on the Nutrition Screening Initiative Checklist: low (59.5%), moderate (23.7%), and high (16.8%) nutritional risk. Significant differences were found between nutritional status and the following four baseline variables: age, hypertension, cerebrovascular diseases, and current smoking. However, no significant differences were evident between nutritional status and sex, body mass index, diabetes, drinking habit, or exercise habit. Logistic regression analysis adjusted for age, sex, body mass index, hypertension, cerebrovascular diseases and smoking habit showed that the high nutritional risk group was significantly associated with a decline in both activities of daily living (odds ratio: 4.96; 95% confidence interval (CI): 1.59–15.50) and instrumental activities of daily living (OR: 2.58; 95% CI: 1.31–5.06) compared with the low nutritional risk group.

**Conclusions:**

Poor nutritional status based on the Nutrition Screening Initiative Checklist was associated with a decline in functional capacity over a 2-year period in community-dwelling Japanese elderly. These results suggest that the Nutrition Screening Initiative Checklist is a suitable tool for predicting functional capacity decline in community-dwelling elderly.

## Introduction

Nutritional well-being is an important determinant of functional capacity, independence, and quality of life for the elderly [1]–[4]. However, nutritional status can easily deteriorate with age due to physiological, psychological, social, and economic changes [5], [6]. Therefore, to maintain appropriate nutritional status and improve quality of life among the elderly, several screening tools have been developed to identify individuals at nutritional risk. The Nutrition Screening Initiative Checklist (NSIC) was published by the American Academy of Family Physicians, the American Dietetic Association, the National Council on the Aging, and others in 1991 [7], [8]. It aims to identify elderly aged 65 and older at nutritional risk for primary health care purposes with the goal of drawing attention to nutritional problems [9]. Presently, the NSIC is widely used as a nutritional screening tool among community-dwelling elderly in the United States of America and other countries; however, its use remains limited in Japan.

Japanese have a long life expectancy (79.44 years for men and 85.90 years for women in 2011), and thus Japan has the highest proportion of elderly in the world (24.1% in 2012) [10]. By 2060, it is estimated that the proportion of elderly in Japan will be 39.9% [10]. As a result, medical expenses and the need for long-term care insurance are expected to increase; therefore, it is very important for elderly to maintain an independent lifestyle. Functional capacity is regarded as an essential component for independent living later in life, [11].

Functional capacity refers to the ability to perform independent living tasks, and consists of activities of daily living (ADL) and instrumental ADL (IADL) [12]. Identifying elderly who are at risk of diminished functional capacity at an early stage can lead to interventions to prevent further functional capacity decline; therefore, creating a tool for predicting functional capacity decline would be useful.

Some cross-sectional studies have reported an association between nutritional status based on the NSIC and functional capacity decline [13]-[15]. However, no longitudinal studies of the relationship between nutritional status according to the NSIC and functional capacity decline among community-dwelling elderly have been reported.

This study aimed to assess whether nutritional status based on the NSIC is a useful tool for predicting functional capacity decline through a 2-year observational cohort study of Japanese community-dwelling elderly.

## Materials and Methods

### Subjects

Participants were Japanese elderly living in Takatsuki City, an urban area in the northern part of Osaka Prefecture that is home to 90,060 individuals aged 65 and older. In 2013, the proportion of elderly in Takatsuki City was 25.3%. Community and welfare centers for the aged are the main organizations providing social support to the community-dwelling elderly. All participants were recruited through local newspapers and by the local welfare commissioner. A total of 699 elderly subjects aged 65 and older who used or registered at these centers were entered in the baseline survey. The anthropometric measurements of the baseline survey were carried out in May–June of 2006 and 2007. Self-report questionnaires were sent by mail to participants two weeks before the planned collection date, and collected in person on the day the anthropometric measurements were taken.

**Fig 1:**
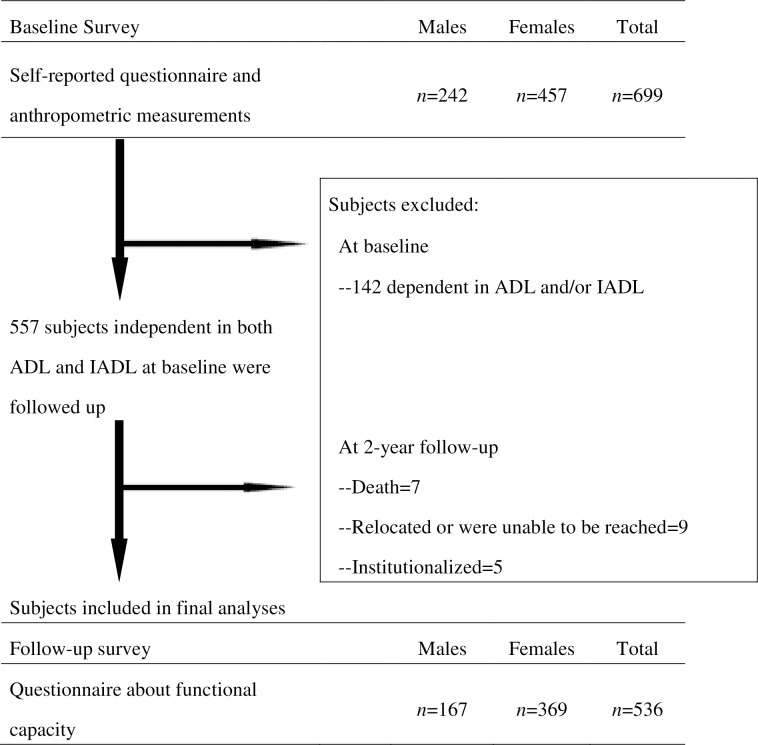
Flowchart of study subjects. ADL, activities of daily living; IADL, instrumental ADL.

To assess whether nutritional status was a predictor of functional capacity decline, the outcome measure was defined as new onset of functional capacity decline. Therefore, we limited the subjects of this study to those who were independent in both ADL and IADL. As a result, 142 subjects who were dependent in ADL and/or IADL in the baseline survey were excluded. The follow-up survey, which was carried out in May–June of 2008 and 2009, consisted of a questionnaire about functional capacity. Of the 557 subjects who were independent in both ADL and IADL, 21 were excluded at the 2-year follow-up for the following reasons: death, had relocated or could not be reached, and institutionalization. Therefore, 536 subjects (167 males, 369 females) were included in the final analyses (Fig 1). We confirmed that the participants had responded to all questions in the baseline and follow-up survey during the collection of the questionnaires.

This study was approved by the Osaka Medical College ethics committee. All subjects provided written informed consent.

### Assessment methods

The baseline survey questionnaire assessed demographic attributes (age and sex), chronic illness (hypertension, diabetes, and cerebrovascular disease), lifestyle-related habits (current drinking, smoking, and exercise status), nutritional status, and functional capacity. Drinking and smoking status were categorized as current, past, or never drinker/smoker. Body mass index (BMI) was used as the anthropometric measurement. The participants’ body height and weight were measured, and BMI was calculated as weight divided by height squared (kg/m^2^).

### Assessment of nutritional status

**Fig 2:**
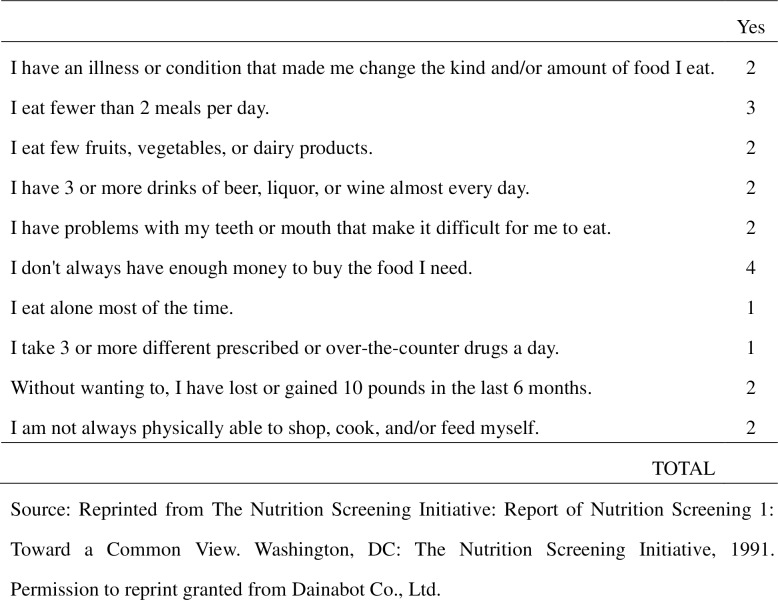
Nutrition Screening Initiative Checklist. Source: Reprinted from The Nutrition Screening Initiative: Report of Nutrition Screening 1: Toward a Common View. Washington, DC: The Nutrition Screening Initiative, 1991. Permission to reprint granted from Dainabot Co., Ltd.

The NSIC is composed of 10 items related to lifestyle and living environment that are considered risk factors for protein-energy malnutrition in the elderly [1], and was used to evaluate nutritional status in this study (Fig 2). Scores for the NSIC items reflect each item’s relative importance as an independent indicator of nutritional risk. Total score (range: 0–21) was used to categorize respondents into three groups based on nutritional risk. Those scoring 0–2 were classified as the “low nutritional risk” group, those scoring 3–5 as the “moderate nutritional risk” group, and those scoring 6 or more as the “high nutritional risk” group [1], [7], [9], [16]. The cut-off points for the low, moderate, and high nutritional risk groups were based on a study by Posner *et al*., who evaluated the effectiveness of the NSIC and determined its sensitivity, specificity, and positive predictive value when used to identify older persons with estimated intakes below 75% of the recommended dietary allowances for three or more nutrients, or those with fair or poor perceived health [1].

### Assessment of functional capacity

Functional capacity was assessed at the baseline and follow-up surveys using both an ADL and an IADL scale. ADL was measured using a modified Katz’s ADL scale, which consists of the following five items: bathing, dressing, walking, continence, and feeding [17]. Subjects who were unable to perform these activities or who needed help were classified as dependent in ADL, while those who were able to perform the activities without assistance were classified as independent in ADL. IADL was measured using a sub-scale for instrumental self-maintenance from the Tokyo Metropolitan Institute of Gerontology Index of Competence (TMIG-IC) [18], [19], which assesses the following five items: using public transportation, shopping for daily necessities, preparing meals, paying bills, and handling a bank account. The response to each item was either “yes” (able to perform the activity, 1 point) or “no” (unable to perform the activity, 0 points) [19]. Subjects whose total score was ≤ 4 were classified as dependent in IADL, while those whose total score was 5 were classified as independent in IADL. We used the TMIG-IC because it was developed for Japanese elderly and has been widely used in the Japanese community [18].

In the follow-up survey, functional capacity was evaluated using ADL and IADL separately; therefore, the categories for the follow-up survey comprised “ADL decline/non-ADL decline” and “IADL decline/non-IADL decline”.

### Statistical analysis

Values were calculated as the mean ± standard deviation for continuous variables and as proportions for categorical variables. Differences in means of variables of subjects based on nutritional status were analyzed using analysis of variance. Differences in proportions of variables of subjects based on nutritional status were analyzed using chi-square tests. Differences in proportions of variables of subjects based on nutritional status between ADL decline and non-ADL decline and between IADL decline and non-IADL decline between baseline and follow-up were analyzed using the chi-square test. Logistic regression analysis, adjusted for age, sex, BMI, hypertension, cerebrovascular diseases, and smoking habit, was used to examine the association between functional capacity decline over 2 years (dependent variable) and baseline nutritional status according to the NSIC (independent variable). Results are presented as odds ratios (ORs) with 95% confidence intervals (CIs). All analyses were performed using the SPSS 19.0 package (SPSS, Chicago, IL). A p-value less than 0.05 was considered statistically significant.

## Results

### Subject characteristics at baseline

Table 1 shows the differences in baseline characteristics among the three groups. Based on the NSIC score, 59.5% of subjects were placed in the low nutritional risk group, 23.7% in the moderate-risk group, and 16.8% in the high-risk group. Significant differences were found between nutritional status and the four baseline variables of age, hypertension, cerebrovascular diseases, and current smoking. On the other hand, no significant differences were apparent in sex, BMI, diabetes, drinking status, and exercise status among the groups at baseline.

**Table 1:** Baseline characteristics of the 536 subjects classified by nutritional status based on the NSIC.

Baseline characteristics	Total	Group			
		Low nutritional risk (*n* = 319)	Moderate nutritional risk (*n* = 127)	High nutritional risk (*n* = 90)	*P*-value
Age (y)	72.9 ± 5.4	71.9 ± 4.8	73.1 ± 5.3	75.9 ± 6.3	<0.0001*
Males (%)	31.2	31.0	29.9	33.3	0.864
BMI (kg/m^2^)	23.0 ± 3.1	23.0 ± 3.2	23.2 ± 2.9	22.8 ± 2.9	0.708
Hypertension (%)	36.2	27.3	50.4	47.8	<0.0001*
Diabetes (%)	9.7	7.5	15.0	10.1	0.053
Cerebrovascular diseases (%)	4.9	3.1	3.9	12.2	0.002*
Current drinker (%)	15.5	13.2	15.7	23.3	0.096
Current smoker (%)	6.0	3.8	3.9	16.7	<0.0001*
Habit of exercise (%)	65.1	67.4	66.1	55.6	0.11

BMI, body mass index; NSIC, Nutrition Screening Initiative Checklist.

*Statistically significant at p < 0.05.

Values are expressed as mean ± standard deviation or proportion (%). Differences in mean variables of subjects based on nutritional status were analyzed using analysis of variance. Differences in proportions of variables of subjects based on nutritional status were analyzed using the chi-square test.

### Nutritional status at baseline and functional capacity at follow-up

Table 2 shows changes in functional capacity between baseline and the two-year follow-up for the three groups as evaluated by the chi-square test. Of the 536 total subjects, 4.5% showed ADL decline and 12.7% showed IADL decline. ADL decline was seen at follow-up in 1.9%, 5.5%, and 12.2% of the low, moderate, and high nutritional risk groups, respectively. Moreover, 8.2%, 13.4%, and 27.8% of the low, moderate, and high nutritional risk groups, respectively, showed IADL decline at follow-up. The percentage of subjects with decline in both ADL and IADL increased significantly as nutritional status worsened.

**Table 2:** Comparison of functional capacity status at follow-up by nutritional status group based on the NSIC among the 536 subjects.

Functional capacity status at follow-up	Total	Group			
		Low nutritional risk (n = 319)	Moderate nutritional risk (n = 127)	High nutritional risk (n = 90)	*P*-value
ADL					
ADL decline [*n* (%)]	24 (4.5)	6 (1.9)	7 (5.5)	11 (12.2)	<0.0001*
Non-ADL decline [*n* (%)]	512 (95.5)	313 (98.1)	120 (94.5)	79 (87.8)	
IADL					
IADL decline [*n* (%)]	68 (12.7)	26 (8.2)	17 (13.4)	25 (27.8)	<0.0001*
Non-IADL decline [*n* (%)]	468 (87.3)	293 (91.8)	110 (86.6)	65 (72.2)	

ADL, activities of daily living; IADL, instrumental ADL; NSIC, Nutrition Screening Initiative Checklist.

*Statistically significant at p < 0.05.

Differences in proportions of variables of subjects based on nutritional status between ADL decline and non-ADL decline and between IADL decline and non-IADL decline at follow-up were analyzed using the chi-square test.

Table 3 shows the association between nutritional status at baseline and functional capacity decline at the 2-year follow-up period as evaluated by logistic regression. The high nutritional risk group was significantly associated with a decline in both ADL (OR: 4.96; 95% CI: 1.59–15.50) and IADL (OR: 2.58; 95% CI: 1.31–5.06) compared with the low nutritional risk group. On the other hand, the declines were not significant between the moderate and the low nutritional risk groups in ADL (OR: 3.00; 95% CI: 0.96–9.35) and IADL (OR: 1.45; 95% CI: 0.74–2.86).

**Table 3:** Association between nutritional status at baseline based on the NSIC and functional capacity decline at the 2-year follow-up among the 536 subjects.

	ADL decline (*n* = 24)/ Non-ADL decline (n = 512)		IADL decline (*n* = 68)/ Non-IADL decline (n = 468)	
	OR (95%CI)	*P* value	OR (95%CI)	*P* value
Group				
Low nutritional risk	1.0 (reference)		1.0 (reference)	
Moderate nutritional risk	3.00 (0.96, 9.35)	0.058	1.45 (0.74, 2.86)	0.279
High nutritional risk	4.96 (1.59, 15.5)	0.006	2.58 (1.31, 5.06)	0.006

NSIC, Nutrition Screening Initiative Checklist; ADL, activities of daily living; IADL, instrumental ADL; OR, odds ratio; CI, confidence interval.

Logistic regression analysis, adjusted for age, sex, body mass index, hypertension, cerebrovascular diseases, and smoking habit and using low nutritional risk as the reference, was used to determine odds ratios and 95% CIs. ADL and IADL at follow-up were assigned as the dependent variable, and baseline nutritional status according to the NSIC was assigned as the independent variable.

## Discussion

In Japan, serum albumin, total cholesterol, dietary variety, and chewing ability have been used as an index of nutritional status among community-dwelling elderly who present with functional capacity decline [20]–[22]. The NSIC was previously criticized for its poor specificity and sensitivity [23]; however, its specificity, validity, and reliability have been confirmed by several recent studies. A study of 1161 subjects, mostly community-dwelling European elderly, reported a similar specificity for high nutritional risk between the NSIC and the Mini Nutritional Assessment (MNA), another tool that has been widely used for nutritional screening [24]. Recently, Phillips et al. reported in a systematic literature review that the NSIC is one of the most extensive screening tools for evaluating nutrition among community-dwelling elderly in terms of validity and reliability [4]. Moreover, the NSIC can be used not only as a screening tool, but also as an educational tool [25]. It includes the risk factors associated with nutritional risk in the elderly, namely disease, eating poorly, tooth loss/mouth pain, economic hardship, reduced social contact, multiple medicines, involuntary weight loss/gain, requiring assistance in self-care, and advanced age [26]; using the NSIC can help the elderly become aware of the warning signs of their nutritional risk. Thus, the NSIC is useful for promoting the nutritional well-being of the elderly in the community setting.

Some cross-sectional studies have shown an association between NSIC-based nutritional status and functional capacity decline [13]-[15]. A study of 2605 community-dwelling elderly in Singapore reported that participants with a high and moderate nutritional risk status, as determined by the NSIC, were 1.72 times more likely than those with low nutritional risk status to report ADL or IADL disability [14]. In addition, we have previously reported that high nutritional risk status as determined by the NSIC was significantly associated with disabilities in higher-level competence, such as IADL, intellectual activity, and social role measured with the TMIG-IC, among community-dwelling Japanese elderly [15]. However, there is no known previous report about the relationship between nutritional status based on the NSIC and functional capacity decline in a cohort of community-dwelling elderly, especially in Japan.

The present study showed that the high nutritional risk group had a significant two-year decline in both ADL (OR: 4.96; 95% CI: 1.59–15.50) and IADL (OR: 2.58; 95% CI: 1.31–5.06) compared with the low nutritional risk group. To the best of our knowledge, this study is the first cohort study to suggest that nutritional status based on the NSIC is a predictor of functional capacity decline in community-dwelling elderly in Japan.

This study had several limitations. First, our subjects were regular attendees of community centers or welfare centers and, therefore, might have been in good health. Our results for the decline in IADL over two years differ from a previous report of 1274 community-dwelling Japanese elderly aged 65 and older which showed a 15.2% IADL decline over 2-years [27]. However, more than 40% of the subjects in this study were classified as moderate or high nutritional risk. It is inferred that the NSIC includes multiple risk factors (e.g., economic situation and poor health) associated with nutritional risk in the elderly. Thus, these community or welfare centers may be good places to introduce future interventions aimed at the comprehensive prevention of poor nutritional status. Second, this study only compared nutritional assessment criteria using BMI and did not assess other anthropometric measurements, such as mid-arm and calf circumference, or dietary and laboratory data. Thus, all findings obtained in this study need to be confirmed by future studies. Third, this study found the NSIC to be a useful tool for predicting functional capacity decline. Future studies are needed to estimate the sensitivity and specificity of the NSIC as a screening tool for functional decline.

In conclusion, this study suggested that nutritional status based on the NSIC is useful for predicting functional capacity decline in community-dwelling Japanese elderly. Interventions to prevent poor nutritional status are very important to prevent functional decline among elderly individuals. The NSIC is easy, noninvasive, inexpensive and does not require anthropometric or biochemical measurements. Thus, it is a suitable tool for public health research among community-dwelling elderly. This study suggests that the NSIC can be widely used to predict functional capacity decline in community-dwelling elderly.
